# The Impact of Electronic Reading Devices on Reading Speed and Comfort in Patients with Decreased Vision

**DOI:** 10.1155/2017/3584706

**Published:** 2017-04-20

**Authors:** Henry L. Feng, Daniel B. Roth, Howard F. Fine, Jonathan L. Prenner, Kunjal K. Modi, William J. Feuer

**Affiliations:** ^1^NJ Retina, Department of Ophthalmology, Rutgers Robert Wood Johnson Medical School, New Brunswick, NJ 08901, USA; ^2^Bascom Palmer Eye Institute, University of Miami Leonard M. Miller School of Medicine, Miami, FL 33136, USA

## Abstract

*Background/Aims*. To evaluate the impact of back-illuminated and nonilluminated electronic reading devices on reading speed and comfort in patients with decreased vision. *Methods*. A prospective study involving a convenience sample of 167 patients at a single retina practice from January 2011 to December 2012. Participants were asked to read five different excerpts on five different media in a randomly assigned order. Media included a printed book at 12-point font (12PF), iPad2 at 12PF, iPad2 at 18-point font (18PF), Kindle2 at 12PF, and Kindle2 at 18PF. Reading speed in words per minute (WPM) and medium preference were recorded and stratified by visual acuity (VA). *Results*. Mean reading speeds in WPM: iPad2 at 18PF (217.0), iPad2 at 12PF (209.1), Kindle2 at 18PF (183.3), Kindle2 at 12PF (177.7), and printed book at 12PF (176.8). Reading speed was faster on back-illuminated media compared to nonilluminated media. Text magnification minimized losses in reading performance with worsening patient VA. The majority of participants preferred reading on the iPad2 at 18PF. *Conclusions*. Back-illuminated devices may increase reading speed and comfort relative to nonilluminated devices and printed text, particularly in patients with decreased VA.

## 1. Introduction

Visual acuity (VA), a fundamental parameter used in the practice of ophthalmology, is typically evaluated through a patient's ability to identify letters and figures on a contrasted background. In many clinical settings, a modified version of the original 1862 Snellen chart is still used to determine a patient's distance VA [1]. As a result, distance VA continues to be assessed regularly in modern clinical practice and is often used to make inferences about a patient's visual functional status. However, near vision is also imperative for daily tasks and maintaining quality of life; one study reported that the most common presenting visual complaint in patients with age-related macular degeneration (AMD) is difficulty with near reading [2].

Prior studies have shown that VA is one of several key factors related to reading performance [3–5]. However, variability in ambient illumination and background contrast may lead to inconsistent measurements of VA [6]. In one example, Falkenstein et al. [7] demonstrated that patients achieved better VA when using the Early Treatment Diabetic Retinopathy Study (ETDRS) chart, which has a background luminance of 121.0 cd/m^2^, than when using the standard Snellen chart, which has a background luminance of 65.0 cd/m^2^. Similarly, our prior study found that near VA was approximately 1 line better when measured on a back-illuminated electronic device than when measured on an equivalent Rosenbaum near vision card [8]. As a result, back-illumination may be one strategy for improving VA and reading performance.

Given the widespread prevalence of macular disease and growing number of low-vision patients, it is becoming progressively more essential that effective visual aids are developed and accessible [9]. Over the past several years, tablets, smart phones, and other electronic devices have become not only more accessible to the general public but also more diverse in their applications [10]. Although the previous studies have implemented standardized reading texts in order to objectively assess reading performance, many of these studies employ only printed reading media [11, 12]. Thus, it remains unclear whether different reading media, such as electronic devices, may increase reading speed and comfort. This study aims to evaluate the ability of back-illuminated and nonilluminated electronic reading devices to enhance reading speed and comfort.

## 2. Methods

### 2.1. Participants

This IRB-approved prospective study included a convenience sample of 167 patients from a single retina practice enrolled from January 2011 to December 2012. Subjects were included in the study if they were at least 18 years of age, native English speakers, able to see printed text at 12-point font on a white sheet of a printer paper, and able to read out loud at the eighth grade vocabulary level. All participants provided oral informed consent and completed the study protocol prior to undergoing pupillary dilation and ophthalmic examination. This study complied with the Health Insurance Portability and Accountability Act of 1996 and followed the tenets of the Declaration of Helsinki.

### 2.2. Reading Material

Because equivalent versions of the Minnesota Low-Vision Reading Test (MNREAD) [11] and International Reading Speed Texts (IReST) [12] were not yet standardized on electronic media at the time this study was conducted, five individual excerpts with similar vocabulary and word length were preselected from Mitch Albom's *The Five People You Meet in Heaven*. Each excerpt was randomly presented on 1 of 5 reading media, including a printed book with 12-point font (12PF), an iPad2™ (back-illuminated device, screen size 7.9″ diagonal, Apple Computers, Cupertino, CA) at 12PF, an iPad2 at 18-point font (18PF), a Kindle2™ (nonilluminated device, Amazon, Seattle, WA) at 12PF, and a Kindle2 at 18PF. The text on the iPad2 was rendered using the Amazon Kindle application at 100% screen brightness.

### 2.3. Reading Protocol

All study procedures were conducted in a standard, well-lit clinical examination room. Each participant was trained and given an opportunity to practice page turning on both the iPad2 and Kindle2 prior to the start of testing. Participants were then asked to read each of the five preselected excerpts in a randomly assigned order on randomly selected media. Participants were timed for one minute of reading on each medium, for a total reading time of five minutes. Each participant was instructed to wear corrective lenses, keep both eyes open, and read out loud at a comfortable volume. Immediately upon completion of all reading tasks, participants were asked, “Which of the five reading media felt most comfortable for you?”

### 2.4. Main Outcomes and Measures

Reading speed was calculated in words per minute (WPM), and reading preference was recorded based on each participant's response to the question above. Clinical data collected include age, gender, VA, presence or absence of macular disease, specific macular diagnosis, and lens status. Associated macular diagnoses included age-related macular degeneration (AMD), diabetic macular edema (DME), cystoid macular edema (CME), and epiretinal membrane (ERM). Lens statuses included clear, any degree of any cataract, and posterior chamber intraocular lens (PCIOL).

### 2.5. Statistical Analysis

Statistical analyses were carried out using Statistical Analysis System (SAS Institute, Cary, North Carolina). In order to correlate reading speed with VA, participants were stratified into VA groups based on best-corrected visual acuity (BCVA) of the better eye. VA groups included those with good VA (20/20 to 20/25), moderate VA (20/30 to 20/40), poor VA (20/50 to 20/80), and very poor VA (20/100 or worse). Repeated measures ANOVA with post hoc least significant difference (LSD) analysis was used to compare differences in mean reading speeds among VA groups. Chi-squared tests were used to compare differences in preferred reading media among VA groups.

## 3. Results

The present study included 167 participants (69 male, 98 female) with a mean age of 73.5 years (SD = 14.5, range 24 to 95). Median BCVA of the better eye was 20/30, with a range of 20/20 to 9/200. When stratified into VA groups based on BCVA of the better eye, 36% had good VA (20/20 to 20/25), 42% had moderate VA (20/30 to 20/40), 16% had poor VA (20/50 to 20/80), and 7% had very poor VA (20/100 or worse). Macular disease (AMD, DME, CME, or ERM) was present in the better eye in 67% of participants. 3% of participants had clear lenses in the better eye, 53% had at least trace cataracts, and 44% had a PCIOL.

**Figure 1:**
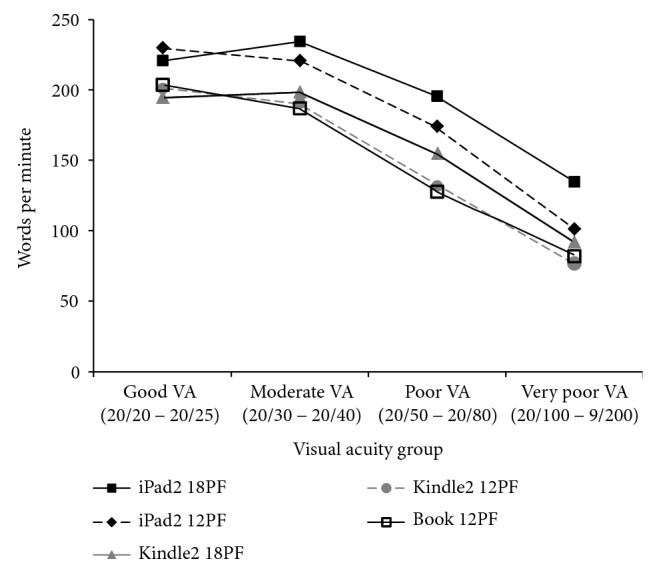
Reading speed stratified by the visual acuity group and reading medium. Mean reading speed in words per minute (WPM) for each reading medium at 12-point font (12PF) or 18-point font (18PF), stratified by the visual acuity (VA) group.

Overall, participants read fastest on the iPad2 at 18PF and slowest on the printed book (Table 1). Post hoc LSD analysis revealed that mean reading speeds were faster on the iPad2 at both font sizes than on the book and Kindle2 at both font sizes (*p* < 0.001). However, there was no significant difference in mean reading speed among the book, Kindle2 at 12PF, and Kindle2 at 18PF (*p* = 0.066). Participants read significantly faster on the iPad2 at 18PF than on the iPad2 at 12PF (*p* = 0.040). When stratified by VA, the iPad2 at both font sizes outperformed the other 3 media across all VA groups (Table 2, Figure 1). However, in contrast to overall results, the subgroup of participants with good VA demonstrated a higher mean reading speed on the iPad2 at 12PF (230.1 WPM) than on the iPad2 at 18PF (220.9 WPM). Lastly, the rate of decline in reading speed with worsening VA was less prominent on 18PF media (iPad2 at 18PF and Kindle2 at 18PF) than on 12PF media (book, iPad2 at 12PF, and Kindle2 at 12PF, *p* = 0.023, Greenhouse-Geisser-corrected repeated measures analysis of covariance).

Among all participants, 69% preferred reading on the iPad2 at 18PF, 23% preferred the Kindle2 at 18PF, 5% preferred the book, 2% preferred the iPad2 at 12PF, and 1% preferred the Kindle2 at 12PF (Table 1). In each VA group, the majority of participants also preferred reading on the iPad2 at 18PF; however, there was no significant association between reading medium preference and the VA group (*p* = 0.33). However, analyzing the 3 media with 12PF as a single group (book, iPad2 at 12PF, and Kindle2 at 12PF) revealed that the iPad2 at 18PF was increasingly preferred with worsening VA (*p* = 0.008, exact Kruskal-Wallis test).

## 4. Conclusions

Although poor VA was defined as 20/50 or worse in this study, the World Health Organization groups those with moderate and severe visual impairment under the term low vision. However, colloquially low vision is often used as a generic term to describe difficulty in performing an array of vision tasks that patients may identify with, including driving, watching television, identifying objects, and reading. While magnifying glasses and other lens-oriented devices have been widely used to improve near reading, these aids can be cumbersome and may not provide the desired level of satisfaction or ease of reading. The use of back-illuminated electronic reading devices is a novel approach to enhancing reading speed and comfort in low-vision patients.

Overall, our results suggest that patients with varying degrees of visual impairment may benefit from the use of back-illuminated electronic reading devices when attempting reading tasks. Participants in this study read faster on a back-illuminated device than on a printed book or nonilluminated device. In fact, mean reading speed was significantly faster on a back-illuminated device with regular font (209.1 WPM) than on a nonilluminated device with magnified font (183.3 WPM). The advantage of back-illumination was even more pronounced in participants with lower VA (Table 2, Figure 1). Given that reading performance has been shown to be a function of multiple visual parameters such as VA, contrast sensitivity, and fixation stability, this improvement in reading speed may, in part, be due to the ability of back-illuminated devices to enhance text contrast and VA, especially in those with impaired visual function [3–5, 8].

Participants also read faster with magnified text on both back-illuminated and nonilluminated devices. The exception was in those with good VA, who actually read faster on the iPad2 at 12PF than on the iPad2 at 18PF. However, this finding is reasonable, as those with good VA are likely able to view both regular and magnified fonts with similar clarity, and the larger font only takes longer to saccade over. Notably, decreases in reading speed with worsening VA were less pronounced on 18PF media than on 12PF media, suggesting that text magnification may preserve reading performance in those with vision loss, independent of back-illumination. Accordingly, the majority of participants felt most comfortable reading on a back-illuminated device with magnified font, and this preference became more prominent among those with lower VA.

A significant limitation in this study is that standardized reading texts such as the IReST and MNREAD were not used to assess reading performance because they were not yet adapted onto digital media at the time this study was performed. In order to minimize differences in reading speed due to possible differences in passage difficulty, both the order of passages and reading media were randomized for each participant in this study. With enough participants, each of the 5 passages would theoretically appear on each of the 5 media with similar frequencies, minimizing the confounding effect of passage difficulty on reading speed.

Collectively, our findings demonstrate that while text magnification may be of some benefit in minimizing losses in reading performance with worsening VA, back-illumination significantly improves reading speed and comfort, particularly in patients with decreased vision. As portable electronic devices become more versatile, both medical professionals and patients will continue to adopt them in a variety of healthcare scenarios [13–16]. Electronic reading devices are particularly promising since they may easily magnify text and enhance contrast without cumbersome equipment or significant effort from the user. Additional efforts should be directed towards adapting standardized reading texts onto digital media in order to better assess reading performance on electronic devices. Further studies should also aim to explore other potential applications of electronic devices as visual aids for patients with impaired visual function.

## Figures and Tables

**Table 1:** Reading medium preferences and mean reading speed.

Medium	Mean WPM	SD	Preferred medium
Book	176.8	102.7	5%
iPad2 12PF	209.1	113.3	2%
iPad2 18PF	217.0	104.7	69%
Kindle2 12PF	177.7	94.4	1%
Kindle2 18PF	183.3	82.0	23%

Mean reading speed in words per minute (WPM), standard deviation (SD), and preferred reading medium at 12-point font (12PF) or 18-point font (18PF).

**Table 2:** Visual acuity group and mean reading speed.

Visual acuity group	*N*	Book 12PF	iPad2 12PF	iPad2 18PF	Kindle2 12PF	Kindle2 18PF
Good VA (20/20–25)	60	203.6	230.1	220.9	201.0	194.7
Moderate VA (20/30–40)	70	186.9	220.9	234.5	190.6	198.5
Poor VA (20/50–80)	26	127.7	174.3	195.6	131.7	154.5
Very poor VA (20/100–9/200)	11	81.8	101.3	134.7	76.6	91.8

Mean reading speed in words per minute (WPM) for each reading medium at 12-point font (12PF) or 18-point font (18PF), stratified by the visual acuity (VA) group.
